# Navigating barriers to affording and obtaining insulin and diabetes supplies

**DOI:** 10.1111/1753-0407.13344

**Published:** 2022-12-23

**Authors:** Annabelle E. Wilcox, Kasia J. Lipska, Stuart A. Weinzimer, Jasmine Gujral, Andrew Arakaki, Linda Kerandi, Laura M. Nally

**Affiliations:** ^1^ Physician Associate Program Yale School of Medicine New Haven Connecticut USA; ^2^ Department of Internal Medicine Yale School of Medicine New Haven Connecticut USA; ^3^ Department of Pediatrics Yale School of Medicine New Haven Connecticut USA; ^4^ Yale School of Public Health New Haven Connecticut USA; ^5^ Frank H. Netter School of Medicine New Haven Connecticut USA

## Abstract

**Highlights**

- Our study suggests that people with diabetes (PWD) face issues of affording and obtaining insulin and diabetes supplies, even in a population predominantly on private health insurance.
- Financially independent young adults reported increased compensatory strategies and resulting perilous behaviors to ration or obtain insulin and supplies, indicating that additional issues may arise once transitioning into adulthood.
- This study suggests that improved access and affordability of insulin and diabetes supplies is needed to reduce the financial burden and prevent adverse outcomes among PWD.

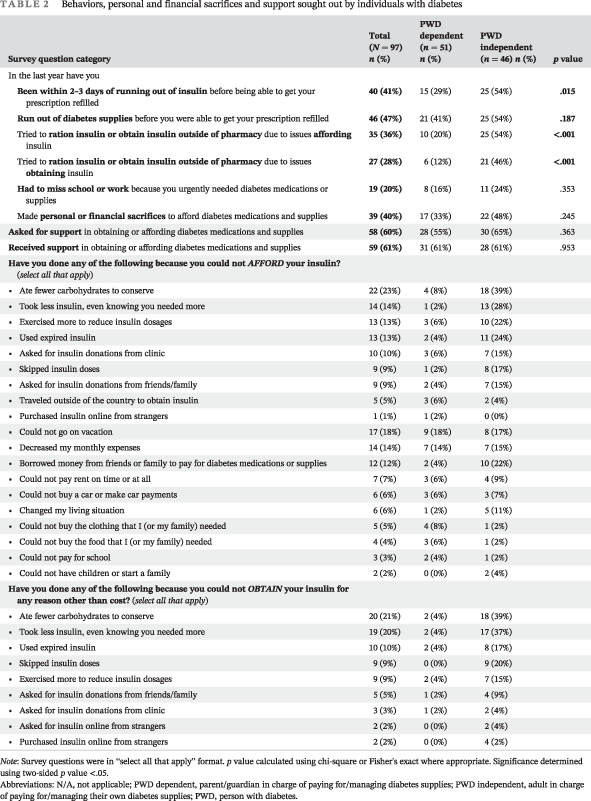

## 1 TO THE EDITOR

The surging costs of insulin and diabetes supplies have pushed people with diabetes (PWD) into unsafe compensatory behaviors.^1^, ^2^, ^3^, ^4^ Data are lacking regarding the manners in which issues of affordability and access change from childhood to adulthood.^5^ We aim to describe difficulties in affording and obtaining insulin and diabetes supplies and compare barriers and coping strategies between children and young adults.

## 2 METHODS

Participants were recruited from the pediatric and adult diabetes clinics at the Yale Diabetes Center and through diabetes‐focused social media groups (Greater Hartford T1D Adult Support Group, Connecticut Chapter of T1International) between April 2020 and January 2021. Participants were required to be a PWD or caregiver of a PWD between the ages of 1 and 41 years, being the primary person in charge of managing and ordering diabetes supplies (on behalf of themselves or another individual), and residing in the state of Connecticut. Participants were classified into two groups based on whether their prescriptions and health insurance were managed independently (PWD independent) or by a parent/guardian (PWD dependent).

The online survey consisted of 87 questions that were grouped into four major categories for analyses: (a) access and affordability of medications and supplies; (b) measures to ration, conserve, or obtain insulin outside of the prescribing pharmacy; (c) personal and or financial sacrifices to afford or obtain medications and supplies; and (d) asking for and/or receiving support in affording or obtaining diabetes medications and supplies.

## 3 RESULTS

Of the 97 responses included for analysis, 47% were in the PWD independent group and 53% were in the PWD dependent group. The population was predominantly non‐Hispanic (88%), white (88%), and female (77%), and most respondents had type 1 diabetes (96%) (Table 1). There was a wide range of educational attainment in both groups, with the PWD independent group reporting lower income more frequently (*p* = .027) (Table 1). Most of the participants had private health insurance (77%) (Table 1); however, 30% lacked insurance in the past, which was more common in the PWD independent group (48%) than the PWD dependent group (14%, *p* < .001). Reported reasons for lack of insurance included “loss of job” and “couldn't afford it.” Regarding supplies, 47% of respondents ran out of supplies before being able to refill their prescription and 41% reported having been within 2–3 days of running out of insulin before being able to refill their prescription. This was significantly more common among the PWD independent group (*p* = .015).

**TABLE 1:** Demographics of the survey respondents

Characteristics	Total (*n* = 97) *n* (%)	PWD dependent (*n* = 51) *n* (%)	PWD independent (*n* = 46) *n* (%)	*p* value
**Type of distribution**				.296
Email through clinic database	77 (79%)	43 (84%)	34 (74%)	
Anonymous link through social media	20 (21%)	8 (16%)	12 (26%)	
**Type of diabetes**				**.021**
Type 1 diabetes	93 (96%)	51 (100%)	42 (91%)	
Type 2 diabetes	4 (4%)	0 (0%)	4 (9%)	
**Age of person filling out survey (mean ± SD)**	35.26 ± 11.53	39.26 ± 13.86	30.83 ± 5.66	**<.001**
**Age of PWD (mean ± SD)**	22.2 ± 9.7	14.3 ± 4.1	30.8 ± 5.7	**<.001**
**Gender of PWD**				**.015**
Female	75 (77%)	46 (90%)	29 (63%)	
Male	21 (22%)	4 (8%)	17 (37%)	
**Ethnicity**				.104
Hispanic/Latinx	7 (7%)	5 (10%)	2 (4%)	
Not Hispanic/Latinx	85 (88%)	43 (84%)	42 (91%)	
Prefer not to answer	5 (5%)	3 (6%)	2 (4%)	
**Race** (select all that apply)				.117
White	85 (88%)	42 (82%)	43 (94%)	
Non‐white (Black or African American, Asian, or other)	4 (4%)	1 (2%)	3 (7%)	
Prefer not to answer	12 (12%)	10 (20%)	2 (4%)	
**Household income**				**.027**
Less than $25 000	10 (10%)	5 (10%)	5 (11%)	
$25 000 to less than $50 000	13 (13%)	5 (10%)	8 (17%)	
$50 000 to less than $75 000	10 (10%)	2 (4%)	8 (17%)	
$75 000 to less than $100 000	12 (12%)	4 (8%)	8 (17%)	
$100 000 to less than $200 000	25 (26%)	14 (28%)	11 (24%)	
$200 000 or more	15 (16%)	12 (24%)	3 (7%)	
Unknown/prefer not to answer	12 (12%)	9 (18%)	3 (7%)	
**Highest level of education**				.641
12th grade or below (no diploma)	2 (2%)	2 (4%)	0 (0%)	
High school diploma or GED	7 (7%)	5 (10%)	2 (4%)	
Some college but no degree	19 (20%)	8 (16%)	11 (24%)	
Associate degree (AA)	7 (7%)	3 (6%)	4 (9%)	
Bachelor's degree (BS, BA, AB)	27 (28%)	12 (24%)	15 (33%)	
Master's degree (MA, MS, MSW, MBA)	24 (25%)	14 (28%)	10 (22%)	
Professional degree (MD, DDS, DVM, JD) or Doctorate degree (PhD, EdD)	7 (7%)	4 (8%)	3 (7%)	
Prefer not to answer	4 (4%)	3 (6%)	1 (2%)	
**Type of health insurance** (select all that apply)				.308
Private health insurance	75 (77%)	41 (80%)	34 (74%)	
Medicaid (Husky)	16 (17%)	9 (18%)	7 (15%)	
Military	2 (2%)	1 (2%)	1 (2%)	
Other state‐sponsored health plan	2 (2%)	2 (4%)	0 (0%)	
Unknown	1 (1%)	0 (0%)	1 (2%)	

*Note*: *p* value calculated using two‐sided *t* test (age), chi‐square (type of distribution) or Fisher's exact (type of diabetes, gender, ethnicity, race, household income, highest level of education, type of insurance status) where appropriate. Significance determined using two‐sided *p* value < .05. *p*‐values < 0.05 were bolded, as they represent statistically significant values.

Abbreviations: person filling out survey, Primary person in charge of paying for/managing diabetes supplies; PWD dependent, parent/guardian in charge of paying for/managing diabetes supplies; PWD independent, adult in charge of paying for/managing their own diabetes supplies; PWD, person with diabetes.

Thirty‐six percent of participants reported using at least one measure to ration insulin or obtain insulin outside of their prescribing pharmacy due to insulin affordability; this was more than twice as common in the PWD independent group (*p* < .001) (Table 2). Overall, 76% of the PWD independent group encountered issues with both insulin affordability and access, leading to compensatory mechanisms to conserve or find insulin other ways (Table 2). Specifically, 61% of the survey respondents reported receiving some financial support from outside sources to obtain supplies (Table 2). Furthermore, 40% of the participants reported making personal or financial sacrifices to afford their insulin and diabetes supplies (Table 2). Again, this was more common in the PWD independent (48%) than the dependent group (33%), though the difference did not reach statistical significance (Table 2).

**TABLE 2:** Behaviors, personal and financial sacrifices and support sought out by individuals with diabetes

Survey question category	Total (*N* = 97) *n* (%)	PWD dependent (*n* = 51) *n* (%)	PWD independent (*n* = 46) *n* (%)	*p* value
In the last year have you				
**Been within 2–3 days of running out of insulin** before being able to get your prescription refilled	**40 (41%)**	15 (29%)	25 (54%)	**.015**
**Run out of diabetes supplies** before you were able to get your prescription refilled	**46 (47%)**	21 (41%)	25 (54%)	**.187**
Tried to **ration insulin or obtain insulin outside of pharmacy** due to issues **affording** insulin	**35 (36%)**	10 (20%)	25 (54%)	**<.001**
Tried to **ration insulin or obtain insulin outside of pharmacy** due to issues **obtaining** insulin	**27 (28%)**	6 (12%)	21 (46%)	**<.001**
**Had to miss school or work** because you urgently needed diabetes medications or supplies	**19 (20%)**	8 (16%)	11 (24%)	.353
Made **personal or financial sacrifices** to afford diabetes medications and supplies	**39 (40%)**	17 (33%)	22 (48%)	.245
**Asked for support** in obtaining or affording diabetes medications and supplies	**58 (60%)**	28 (55%)	30 (65%)	.363
**Received support** in obtaining or affording diabetes medications and supplies	**59 (61%)**	31 (61%)	28 (61%)	.953
**Have you done any of the following because you could not *AFFORD* your insulin?** (*select all that apply*)				
Ate fewer carbohydrates to conserve	22 (23%)	4 (8%)	18 (39%)	
Took less insulin, even knowing you needed more	14 (14%)	1 (2%)	13 (28%)	
Exercised more to reduce insulin dosages	13 (13%)	3 (6%)	10 (22%)	
Used expired insulin	13 (13%)	2 (4%)	11 (24%)	
Asked for insulin donations from clinic	10 (10%)	3 (6%)	7 (15%)	
Skipped insulin doses	9 (9%)	1 (2%)	8 (17%)	
Asked for insulin donations from friends/family	9 (9%)	2 (4%)	7 (15%)	
Traveled outside of the country to obtain insulin	5 (5%)	3 (6%)	2 (4%)	
Purchased insulin online from strangers	1 (1%)	1 (2%)	0 (0%)	
Could not go on vacation	17 (18%)	9 (18%)	8 (17%)	
Decreased my monthly expenses	14 (14%)	7 (14%)	7 (15%)	
Borrowed money from friends or family to pay for diabetes medications or supplies	12 (12%)	2 (4%)	10 (22%)	
Could not pay rent on time or at all	7 (7%)	3 (6%)	4 (9%)	
Could not buy a car or make car payments	6 (6%)	3 (6%)	3 (7%)	
Changed my living situation	6 (6%)	1 (2%)	5 (11%)	
Could not buy the clothing that I (or my family) needed	5 (5%)	4 (8%)	1 (2%)	
Could not buy the food that I (or my family) needed	4 (4%)	3 (6%)	1 (2%)	
Could not pay for school	3 (3%)	2 (4%)	1 (2%)	
Could not have children or start a family	2 (2%)	0 (0%)	2 (4%)	
**Have you done any of the following because you could not *OBTAIN* your insulin for any reason other than cost?** (*select all that apply*)				
Ate fewer carbohydrates to conserve	20 (21%)	2 (4%)	18 (39%)	
Took less insulin, even knowing you needed more	19 (20%)	2 (4%)	17 (37%)	
Used expired insulin	10 (10%)	2 (4%)	8 (17%)	
Skipped insulin doses	9 (9%)	0 (0%)	9 (20%)	
Exercised more to reduce insulin dosages	9 (9%)	2 (4%)	7 (15%)	
Asked for insulin donations from friends/family	5 (5%)	1 (2%)	4 (9%)	
Asked for insulin donations from clinic	3 (3%)	1 (2%)	2 (4%)	
Asked for insulin online from strangers	2 (2%)	0 (0%)	2 (4%)	
Purchased insulin online from strangers	2 (2%)	0 (0%)	4 (2%)	

*Note*: Survey questions were in “select all that apply” format. *p* value calculated using chi‐square or Fisher's exact where appropriate. Significance determined using two‐sided *p* value <.05. *p*‐values < 0.05 were bolded, as they represent statistically significant values.

Abbreviations: N/A, not applicable; PWD dependent, parent/guardian in charge of paying for/managing diabetes supplies; PWD independent, adult in charge of paying for/managing their own diabetes supplies; PWD, person with diabetes.

## 4 COMMENTS

Our study highlights the growing financial pressures and barriers to access that PWD are faced with. Dangerous compensatory behaviors (rationing), personal or financial sacrifices, and nontraditional methods (trading) were reported, even in a relatively educated, financially secure population with access to health insurance. These barriers and behaviors would be exacerbated in a more vulnerable structurally disadvantaged population.

Of all of the participants that reported issues with affordability and access, these issues and compensatory strategies were significantly higher in PWD transitioning to adulthood, suggesting increased vulnerability at this life stage, specifically with navigating a complex and unaffordable healthcare system. Most independent adults in our sample came very close to running out of insulin or supplies, putting them at an increased risk for diabetic ketoacidosis and death.

Limitations of this study include a small sample size, limited diversity (race, income, insurance, education), exclusion of non‐English speaking participants, technology/internet access requirement, and longer survey length. The participants were also largely people with type 1 diabetes in the state of Connecticut, limiting the generalizability of results. This survey was distributed during the COVID‐19 pandemic, which may have affected an individual's financial situation and their ability to access their providers.^6^ Future studies should focus on causes and solutions to affordability and access issues and how to prevent adverse outcomes in glycemic control, diabetes‐related hospitalizations, and long‐term complications. In conclusion, numerous financial and practical barriers to affording diabetes medications and supplies require our immediate attention and political action, as the lives of young PWD are at stake.

## AUTHOR CONTRIBUTIONS

Laura M. Nally, Stuart A. Weinzimer, Linda Kerandi, and Kasia J. Lipska: Designed the study. Linda Kerandi and Laura M. Nally: Conducted the study. Annabelle E. Wilcox: Collected and analyzed the data and wrote the manuscript.Andrew Arakaki: Assisted with data analysis. Laura M. Nally, Stuart A. Weinzimer, Linda Kerandi, Andrew Arakaki, Jasmine Gujral, and Kasia J. Lipska: Reviewed/edited the manuscript.

## FUNDING INFORMATION

This research did not receive any specific grant from funding agencies in the public, commercial, or not‐for‐profit sectors.

## DISCLOSURES

Stuart A. Weinzimer reports receiving honoraria from the following entities: Abbott, Dexcom, Insulet, and Tandem (Speaker); Zealand (Consultant). Kasia J. Lipska receives support from the Centers for Medicare and Medicaid Services (CMS) to develop and review publicly reported quality measures and grant support from the National Institutes of Health. Laura M. Nally reported receiving product support from Dexcom as well as salary support from Novo Nordisk and the National Institutes of Health outside the submitted work.

## INSTITUTIONAL REVIEW BOARD

This study was approved by the Yale University Human Investigation Committee review board. E‐consent was obtained before the survey. Survey respondents were deidentified for analysis and reporting.

## References

[1] Herkert D , Vijayakumar P , Luo J , et al. Cost‐related insulin underuse among patients with diabetes. JAMA Intern Med. 2019;179(1):112‐114. 30508012 10.1001/jamainternmed.2018.5008 PMC6583414

[2] Willner S , Whittemore R , Keene D . "Life or death": experiences of insulin insecurity among adults with type 1 diabetes in the United States. SSM Popul Health. 2020;11:100624. 32676533 10.1016/j.ssmph.2020.100624 PMC7352063

[3] Litchman ML , Oser TK , Wawrzynski SE , Walker HR , Oser S . The underground exchange of diabetes medications and supplies: donating, trading, and borrowing, oh my! J Diabetes Sci Technol. 2020;14(6):1000‐1009. 10.1177/1932296819888215 PMC7645126 31801370

[4] Cefalu WT , Dawes DE , Gavlak G , et al. Insulin access and affordability working group: conclusions and recommendations. Diabetes Care. 2018;41(6):1299‐1311. 29739814 10.2337/dci18-0019 PMC13060039

[5] McCoy RG , Kidney RSM , Holznagel D , Peters T , Madzura V . Challenges for younger adults with diabetes. Minn Med. 2019;102(2):34‐36. 31889734 PMC6936754

[6] Czeisler M , Barrett CE , Siegel KR , et al. Health care access and use among adults with diabetes during the COVID‐19 pandemic ‐ United States, February‐march 2021. MMWR Morb Mortal Wkly Rep. 2021;70(46):1597‐1602. 34793416 10.15585/mmwr.mm7046a2 PMC8601412

